# Risk Factors for Pre-Treatment Mortality among HIV-Infected Children in Rural Zambia: A Cohort Study

**DOI:** 10.1371/journal.pone.0029294

**Published:** 2011-12-21

**Authors:** Catherine G. Sutcliffe, Janneke H. van Dijk, Bornface Munsanje, Francis Hamangaba, Pamela Siniwymaanzi, Philip E. Thuma, William J. Moss

**Affiliations:** 1 Department of Epidemiology, Bloomberg School of Public Health, Johns Hopkins University, Baltimore, Maryland, United States of America; 2 Macha Research Trust, Macha Hospital, Choma, Zambia; University of Cape Town, South Africa

## Abstract

**Background:**

Many HIV-infected children in sub-Saharan Africa enter care at a late stage of disease. As preparation of the child and family for antiretroviral therapy (ART) can take several clinic visits, some children die prior to ART initiation. This study was undertaken to determine mortality rates and clinical predictors of mortality during the period prior to ART initiation.

**Methods:**

A prospective cohort study of HIV-infected treatment-naïve children was conducted between September 2007 and September 2010 at the HIV clinic at Macha Hospital in rural Southern Province, Zambia. HIV-infected children younger than 16 years of age who were treatment-naïve at study enrollment were eligible for analysis. Mortality rates prior to ART initiation were calculated and risk factors for mortality were evaluated.

**Results:**

351 children were included in the study, of whom 210 (59.8%) were eligible for ART at study enrollment. Among children ineligible for ART at enrollment, 6 children died (mortality rate: 0.33; 95% CI:0.15, 0.74). Among children eligible at enrollment, 21 children died before initiation of ART and their mortality rate (2.73 per 100 person-years; 95% CI:1.78, 4.18) was significantly higher than among children ineligible for ART (incidence rate ratio: 8.20; 95% CI:3.20, 24.83). In both groups, mortality was highest in the first three months of follow-up. Factors associated with mortality included younger age, anemia and lower weight-for-age z-score at study enrollment.

**Conclusions:**

These results underscore the need to increase efforts to identify HIV-infected children at an earlier age and stage of disease progression so they can enroll in HIV care and treatment programs prior to becoming eligible for ART and these deaths can be prevented.

## Introduction

Since 2003, antiretroviral treatment (ART) programs in sub-Saharan Africa have rapidly scaled-up and currently provide treatment to an estimated 356,000 HIV-infected children [1]. Reports from programs indicate that treatment outcomes among children are comparable to those observed in resource-rich settings [2]. However, many children enter care at a late stage of disease progression, such that mortality rates during the first few months of treatment are high [2]. The period between enrollment into care and treatment initiation can extend for several months as eligible children and their families are prepared for the burden and challenges of ART [3]. This process can involve several clinic and home visits, psychological assessments, ART literacy training, adherence counseling, and identification of a family member or friend to provide support (‘medicine companion’ or ‘treatment supporter’) [4], [5], [6], [7]. During this time, children also are diagnosed and treated for any concurrent opportunistic infections. In addition, children not eligible for ART at enrollment are monitored, typically every three months, and may experience rapid disease progression. Few programs report outcomes during this period prior to ART initiation, but several studies among adults [5], [7], [8], [9], [10], [11] and children [3], [6], [12], [13] indicate that significant mortality occurs among both ART eligible and ineligible individuals during this time of preparation and evaluation.

This study was undertaken to determine mortality rates and identify clinical predictors of mortality during the period prior to ART initiation among both eligible and ineligible children enrolled in an ART program in rural Zambia.

## Methods

### Ethics Statement

The study was approved by the Government of Zambia Ministry of Health, the Research Ethics Committee of the University of Zambia and the Institutional Review Board of the Johns Hopkins Bloomberg School of Public Health. Written informed consent was obtained from parents or guardians and assent was obtained from children 8–16 years of age.

### Study Population

The study was conducted at Macha Hospital in a rural area of Southern Province, Zambia. The study setting and population have been described in detail elsewhere [14], [15]. Briefly, Macha Hospital is a district-level referral hospital administered by the Zambian Brethren in Christ Church. Since 2005, Macha Hospital has provided care to over 7000 HIV-infected adults and children through the Government of Zambia's antiretroviral treatment program, with additional support from the President's Emergency Plan for AIDS Relief (PEPFAR) through the non-governmental organization, AidsRelief.

Children with a positive HIV serologic test are referred to the clinic from voluntary counseling and testing programs, outpatient clinics and rural health centers. Early infant diagnosis based on detection of HIV DNA has been available since February 2008. Clinical care is provided without charge by medical doctors and clinical officers, and adherence counseling by nurses and trained counselors. Routine follow up occurs approximately every three months. ART is initiated according to WHO guidelines at the discretion of the doctors and clinical officers. At enrollment in the clinic, children and their caregivers must attend two group counseling sessions to avoid early attrition. Before ART initiation an individual treatment preparation counseling session is provided, and children must demonstrate their ability to adhere to non-ART medications, including multivitamins and cotrimoxazole, over two visits through self-report and pill counts. In addition, individuals must identify a family member or friend to serve as a treatment supporter.

### Study Procedures

Beginning in September 2007, HIV-infected children younger than 16 years seeking HIV care were eligible for enrollment into a cohort study. Children were evaluated at study visits approximately every three months, at which time a questionnaire was administered, the child was examined and a blood specimen was obtained. The study questionnaire was designed to collect information on demographics, the vital status and education of the parents and primary caregiver, the child's medical, medication, and vaccination history, disclosure of HIV status and transportation to the clinic. During each physical examination, height and weight were measured. As part of routine clinical care, CD4^+^ T cell counts and percentages were measured using the Guava Easy CD4 system (Guava Technologies, Inc., Hayward, CA). Hemoglobin was measured using an ABX Micros ES 60 Hematology Analyzer (Horiba Medical, France). For children who missed study visits, home visits were attempted to ascertain their status. For children who died, cause of death was ascertained through verbal autopsy or through hospital or clinic records. Information recorded before study enrollment was abstracted from medical records.

### Statistical Analysis

For the present analysis, all treatment-naïve children enrolled in the study between September 2007 and September 2010 were included. Children were followed until the first of either ART initiation, death, loss to follow-up or September 1, 2010. Children whose last visit occurred more than six months prior to September 1, 2010 were considered lost to follow-up.

Eligibility for ART at study enrollment was defined retrospectively based on the WHO criteria in effect at the time of study enrollment. At the beginning of the study, the 2006 WHO treatment guidelines were in effect [16], which recommended ART for all children with WHO stage 3 or 4 irrespective of immunologic status. For children with WHO stage 1 or 2, ART was recommended on the basis of immunologic status (≤11 months: <25%; 12–35 months: <20%; ≥36 months: 15%). Treatment guidelines were revised in 2008 [17], and ART was recommended for all children younger than 12 months of age. For children older than 12 months, ART initiation was based on clinical (WHO stage 3 or 4) and immunologic (WHO stage 1 or 2 and <20% for children 12–59 months or <15% for children ≥5 years of age) status. The 2008 WHO treatment guidelines were assumed to take effect in June, 2008. For children missing WHO stage or CD4^+^ T-cell percentage at enrollment, ART eligibility was defined based on immunologic or clinical criteria alone. Immunologic and clinical parameters from within three months of study enrollment were used to determine ART eligibility and clinical and immunologic status at study enrollment. Weight-for-age z-scores were calculated based on the WHO growth standards [18] and children with z-scores below −2 and −3 were defined as underweight and severely underweight, respectively. Severe immunodeficiency was defined as CD4^+^ T-cell percentage by age for all children according to the 2006 WHO treatment guidelines (≤11 months: <25%; 12–35 months: <20%; 36–59 months: <15%; ≥5 years: <15%) [16]. Severe anemia was defined as hemoglobin <8 g/dL [19]. A measure of socio-economic status (SES) was calculated based on the Demographic and Health Survey SES scale used in Zambia [20]. SES percentiles were based on the predetermined cutoffs (<25^th^ = 0–6; 26–50^th^ = 7–12; 51–75^th^ = 13–18; >75^th^ = 19–24).

Kaplan-Meier survival methods were used to estimate the cumulative probability of ART initiation and death. Survival curves were compared between groups using the logrank test. Mortality rates were calculated per 100 person-years at risk and were compared using Poisson regression. Risk factors for mortality were evaluated using Cox proportional hazards regression. Characteristics associated with mortality in univariable analysis (p<0.1) were considered for inclusion in the multivariable model. All analyses were conducted in SAS for Windows version 9.2 (SAS Institute Inc., Cary, NC) and Stata, version 11 (StataCorp LP, College Station, Texas).

## Results

During the study period, 363 treatment-naïve HIV-infected children were invited to participate and 362 were enrolled. Three hundred and fifty-one (97%) children had sufficient information to determine their eligibility for ART and were included in the analysis. Children were enrolled in the clinic for a median of 0.20 months (interquartile range [IQR]: 0, 4.16) prior to study enrollment, and the median follow-up time in the study was 3.44 months (IQR: 0.92, 9.44). At study enrollment, the median age was 2.6 years (IQR: 1.4, 5.7), 45.1% of children were male, and 23.5% of children were single or double orphans (Table 1). Over half (54.0%) of children were moderately (24.1%) or severely underweight (30.0%) and the majority had severe disease: 40.3% had severe immunodeficiency, 51.5% were classified as WHO stage 3 or 4, and 59.8% were eligible for ART.

**Table 1:** Baseline characteristics of treatment-naïve, HIV-infected children receiving care at Macha Hospital, Zambia from 2007–2010.

	Study population (n = 351) N (%)
Median age in years (IQR)(n = 351)	2.6 (1.4, 5.7)
<2 yrs	140 (39.9)
2–4 yrs	109 (31.1)
≥5 yrs	102 (29.1)
Male (n = 351)	158 (45.0)
Mother received PMTCT (n = 348)	22 (6.3)
Vital status of parents (n = 347)	
Both alive	264 (76.1)
One parent died	64 (18.4)
Both died	19 (5.5)
Primary caregiver (n = 348)	
Mother/Father	272 (78.2)
Grandparent	39 (11.2)
Aunt/uncle	26 (7.5)
Other	11 (3.2)
Education of primary caregiver (n = 337)	
None	12 (3.6)
Primary	211 (62.6)
Secondary	110 (32.6)
Higher	4 (1.2)
Socioeconomic status (n = 348)	
≤25^th^ percentile	233 (67.0)
26–50^th^ percentile	96 (27.6)
51–75^th^ percentile	17 (4.9)
76–100^th^ percentile	2 (0.6)
Median WAZ (IQR)^a^ (n = 283)	−2.2 (−3.4, −1.3)
≥−2	133 (45.9)
<−2 to −3	70 (24.1)
<−3	87 (30.0)
Median CD4% (IQR) (n = 320)	20.7 (14.7, 27.6)
Severe immunodeficiency^b^	129 (40.3)
WHO stage (n = 264)	
1	50 (18.9)
2	78 (29.6)
3 or 4	136 (51.5)
Eligible for ART^c^ (n = 351)	210 (59.8)

ART: antiretroviral treatment ; IQR: interquartile range; PMTCT: prevention of mother-to-child transmission; WAZ: weight-for-age z-score; WHO: World Health Organization.

a Among children <10 years of age.

b Defined by age for all children according to the 2006 WHO guidelines.

c Defined retrospectively according to the WHO treatment guidelines in effect at the time of study enrollment.

**Figure 1:**
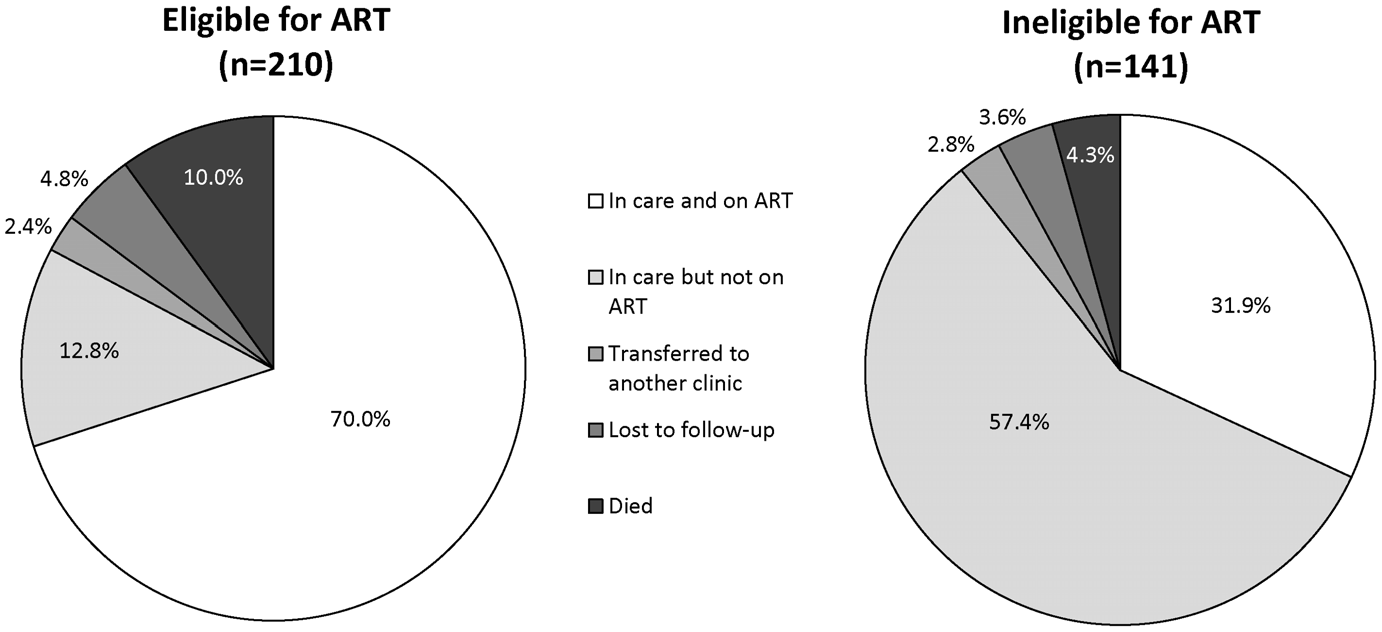
Outcomes at the end of the study period, by eligibility status at study enrollment.

**Figure 2:**
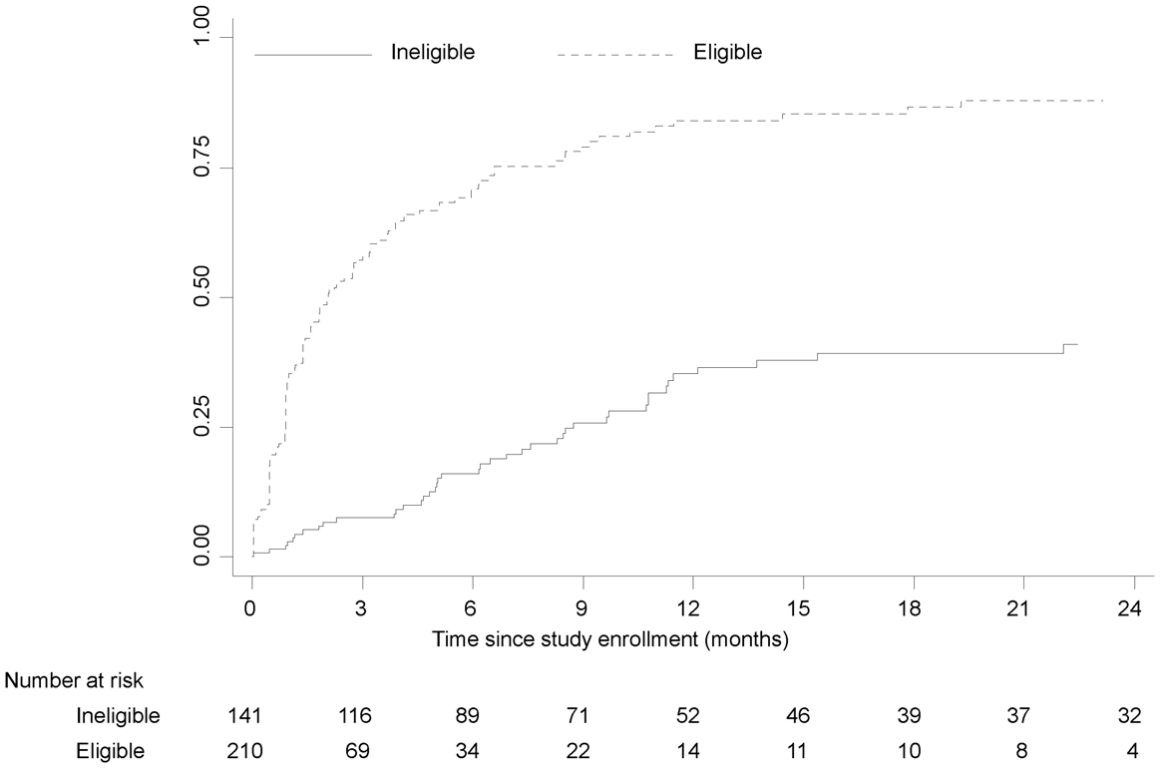
Cumulative probability of ART initiation by eligibility status at study enrollment.

By the end of the study period, 192 (54.7%) children started ART, 15 (4.3%) were lost to follow-up, 9 (2.6%) transferred to another clinic, 27 (7.7%) died, and 108 (30.8%) were still in care but not receiving ART. Children eligible for ART at enrollment were significantly more likely to have started ART (p<0.0001) and died (p = 0.05) (Figure 1). Among children eligible at enrollment, the median time from study enrollment to ART initiation was 2.07 months (IQR: 0.92, 6.59) (Figure 2). At 12 and 24 months, the cumulative probability of ART initiation was 84.1% and 88.0%, respectively. Chart reviews were conducted for 69 children who had either taken more than three months to initiate ART or had been enrolled in the study for more than three months without starting ART to determine reasons for potential delays in ART initiation (Table 2). The majority of children (81.2%) were found to have an identifiable reason for delay. The most common reason was delay on the part of the family (33.9%), due to poor adherence of the child to other medications, family unpreparedness to adhere to the more frequent clinic visit schedule or the child missing clinic visits. Other reasons included provider delay (28.6%), primarily due to misinterpretation of laboratory results, HIV staging or eligibility criteria by clinicians; medical delay (19.6%), primarily due to treatment for tuberculosis; and a combination of family, provider and medical delays (16.1%). Among children ineligible at enrollment, the cumulative probability of ART initiation at 12 and 24 months was 35.3% and 41.0%, respectively (Figure 2).

**Table 2:** Evaluation of delays in ART initiation among 69 children eligible for ART at enrollment who had either taken more than three months to initiate ART or had been enrolled in the study for more than 3 months without starting ART.

	N (%)
**Delayed ART initiation**	**56 (81.2)**
**Family delay**	**19 (33.9)**
Poor adherence	3 (5.4)
Family unpreparedness^a^	4 (7.1)
Family unpreparedness and poor adherence	8 (14.3)
Child stopped coming to the clinic	4 (7.1)
**Provider delay**	**16 (28.6)**
Misinterpretation of laboratory results, HIV staging or eligibility criteria by clinician	10 (17.9)
Other	6 (10.7)
**Medical delay**	**11 (19.6)**
Hepatitis or elevated ALT	1 (1.8)
Tuberculosis	10 (17.9)
**Combined family, provider or medical delay**	**9 (16.1)**
Provider and family delay	6 (10.7)
Tuberculosis and poor adherence	2 (3.6)
Tuberculosis and family unpreparedness	1 (1.8)
**Unknown**	**1 (1.8)**
**No delay**	**13 (18.8)**
Eligible by WHO stage only^b^	10 (76.9)
Other	3 (23.1)
**Total**	**69 (100.0)**

ART: antiretroviral treatment; ALT: alanine aminotransferase; WHO: World Health Organization.

a Family unpreparedness included refusal to come at shorter intervals and problems with transportation.

b Children who are underweight or have specific symptoms and opportunistic infections (e.g. prolonged diarrhea) are classified as WHO stage 3 at that visit. However, they are not considered eligible for ART if their weight or symptoms improve with treatment at subsequent visits.

**Figure 3:**
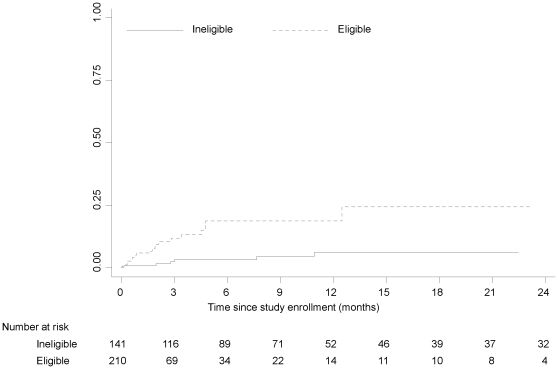
Cumulative probability of mortality by eligibility status at study enrollment.

Among the 27 children who died, the median time from study enrollment to death was 1.90 months (IQR: 0.36, 3.41). Factors contributing to death were available for 25 children and included pneumonia (n = 10), tuberculosis (n = 10), undernutrition (n = 10), diarrhea (n = 8), cerebral malaria (n = 1), meningitis (n = 2), renal failure (n = 1), hepatitis (n = 1) and measles (n = 1). Place of death was available for 21 children; 16 (76.2%) children died in the hospital and 5 (23.8%) children died at home. The cumulative probabilities of death by 6, 12 and 24 months after study enrollment were 9.9% (18.6% eligible, 3.2% ineligible), 12.0% (18.6% eligible, 6.1% ineligible), and 13.4% (24.4% eligible, 6.1% ineligible; logrank test = 0.0003; Figure 3), respectively. The overall pre-ART mortality rate was 1.05 (95% CI: 0.72, 1.53) per 100 person-years. Among children ineligible for ART, the mortality rate was 0.33 (95% CI: 0.15, 0.74) per 100 person-years. Among children eligible for ART, the mortality rate was 2.73 (95% CI: 1.78, 4.18) per 100 person-years and was significantly higher than among children ineligible for ART (incidence rate ratio: 8.20; 95% CI: 3.20, 24.83). The mortality rate was highest in the first three months after study enrollment at 2.58 per 100 person-years (95% CI: 1.65, 4.05), and was higher among children eligible for ART (eligible: 4.50; 95% CI: 2.75, 7.34; ineligible: 0.79; 95% CI: 0.25, 2.44; incidence rate ratio: 5.70; 95% CI: 1.63, 30.55). Twenty-one (77.8%) of the children who died were eligible for ART at study enrollment, and this group was therefore more similar in characteristics at study enrollment to the surviving children eligible for ART (Table 3).

**Table 3:** Comparison of characteristics at study enrollment between children who died, and children who were eligible and ineligible for ART at study enrollment.

	Children who died (n = 27) N (%)	Surviving children eligible for ART^a^ (n = 189) N (%)	p-value^b^	Surviving children ineligible for ART^a^ (n = 135) N (%)	p-value^b^
Male	10 (37.0)	87 (46.0)	0.38	61 (45.2)	0.44
Median age (IQR)	1.4 (0.7, 2.6)	2.0 (1.1, 4.7)	0.07	4.3 (2.1, 7.0)	<0.0001
<2 yrs	18 (66.7)	91 (48.2)		31 (23.0)	
2–4 yrs	5 (18.5)	55 (29.1)		49 (36.3)	
≥5 yrs	4 (14.8)	43 (22.8)	0.20	55 (40.7)	<0.0001
Median WAZ (IQR)^c^	−3.7 (−4.4, −2.1)	−2.3 (−3.6, −1.4)	0.03	−1.8 (−2.6, −0.9)	0.0002
≥−2	4 (21.1)	70 (42.9)		59 (54.6)	
<−2 to −3	4 (21.1)	35 (21.5)		31 (28.7)	
<−3	11 (57.9)	58 (35.6)	0.12	18 (16.7)	0.0003
Median CD4% (IQR)	20.7 (14.9, 26.8)	17.0 (10.5, 21.1)	0.03	25.8 (22.1, 33.3)	0.001
Severe immunodeficiency^d^	10 (41.7)	119 (67.2)	0.01	0 (0.0)	<0.0001
Median hemoglobin (IQR)	8.9 (7.7, 9.3)	9.5 (8.6, 10.5)	0.001	10.6 (9.3, 11.3)	<0.0001
<8 g/dL	8 (33.3)	21 (11.6)	0.004	7 (5.9)	<0.0001
WHO stage					
1	1 (5.3)	11 (7.1)		38 (41.3)	
2	3 (15.8)	21 (13.7)		54 (58.7)	
3 or 4	15 (78.9)	121 (79.1)	0.51	0 (0.0)	<0.0001
Parent's vital status					
Both alive	19 (73.1)	151 (79.9)		94 (71.2)	
One parent died	7 (26.9)	28 (14.8)		29 (22.0)	
Both died	0 (0.0)	10 (5.3)	0.18	9 (6.8)	0.36

ART: antiretroviral treatment; WAZ: weight-for-age z-score; WHO: World Health Organization.

a ART eligibility defined retrospectively according to the WHO treatment guidelines in effect at the time of study enrollment.

b p-value comparing surviving children to children who died.

c Among children <10 years of age.

d Defined by age according to the 2006 WHO guidelines.

**Figure 4:**
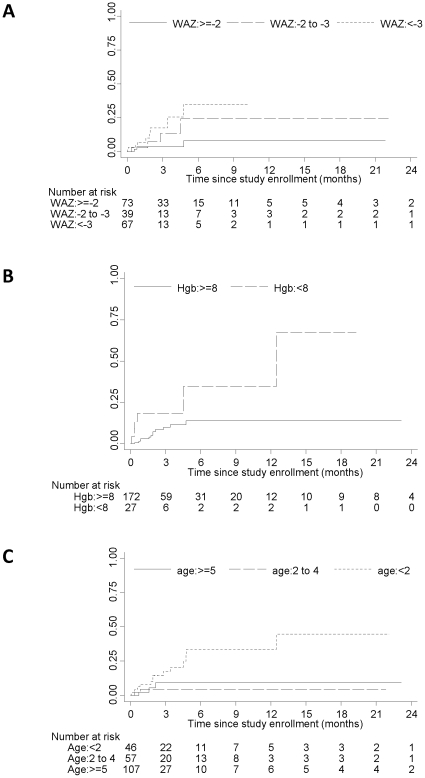
Cumulative probability of mortality by WAZ (A), hemoglobin level (B), and age (C) at study enrollment among children eligible for ART.

Among children eligible for ART at study enrollment, age, WAZ score and anemia were associated with mortality in the crude analysis and were further evaluated. In multivariable analysis, lower WAZ score (HR for <−3 vs. ≥−2: 7.63; 95% CI: 1.80, 32.39) and anemia (HR: 3.50; 95% CI: 1.25, 9.77) remained independently associated with a higher risk of mortality (Table 4). Younger age was marginally independently associated with mortality (hazard rate [HR] for <2 years vs. ≥5 years: 3.58; 95% CI: 0.98, 13.09). CD4^+^ T-cell percentage at study enrollment and sex were not associated with mortality. The cumulative probability of mortality six months after study enrollment was 8.0% for children with WAZ≥−2, 24.3% for children with WAZ −3 to −2, and 35.0% for children with WAZ<−3 (logrank test = 0.04; Figure 4A). The cumulative probability of mortality six months after enrollment was 13.8% for children without anemia and 34.6% for children with anemia (logrank test = 0.001; Figure 4B). The cumulative probability of mortality six months after enrollment was 33.7% for children younger than 2 years of age, 4.5% for children 2 to 4 years of age, and 9.2% for children 5 years of age or older (logrank test = 0.01; Figure 4C). Similar risk factors were found among children who were ineligible for ART at study enrollment, although the number of children and deaths were small and precluded a full evaluation (Table 4).

**Table 4:** Risk factors for mortality among children eligible and ineligible for ART at study enrollment.

	Children eligible for ART (n = 210; 21 deaths)		Children ineligible for ART (n = 141; 6 deaths)
	Crude HR (95% CI)	Adjusted HR (95% CI)	Crude HR (95% CI)
Male	0.53 (0.20, 1.37)	—	2.47 (0.45, 13.49)
Age (years)			
<2	3.31 (0.96, 11.42)	3.58 (0.98, 13.09)	3.90 (0.35, 43.23)
2–4	0.63 (0.11, 3.78)	1.03 (0.16, 6.60)	3.29 (0.34, 31.74)
≥5	1	1	1
Weight-for-age z-score^a^	0.74 (0.54, 1.01)		0.48 (0.26, 0.91)
≥−2	1	1	—
<−2 to −3	2.76 (0.62, 12.34)	4.47 (0.88, 22.74)	—
<−3	4.62 (1.23, 17.28)	7.63 (1.80, 32.39)	—
CD4% (per 10)	1.17 (0.71, 1.95)	—	0.53 (0.16, 1.75)
Severe immunodeficiency^b^	0.92 (0.36, 2.36)	—	—
Hemoglobin <8 g/dL	2.54 (1.48, 4.36)	3.50 (1.25, 9.77)	6.84 (1.25, 37.40)

ART: antiretroviral treatment; HR: hazard ratio.

a Among children <10 years of age.

b Defined by age according to the 2006 WHO guidelines.

## Discussion

Mortality among HIV-infected children in the period prior to ART initiation was high, particularly in the first few months after enrollment among children eligible for ART. Risk factors for mortality included younger age, undernutrition and anemia, and were similar among children eligible and ineligible for ART at study enrollment.

Most treatment programs report on mortality and treatment outcomes after initiation of ART and few have focused on the period prior to ART initiation. However, this period should be evaluated along with the interval after ART initiation as it provides additional insight into the population of children reached by the program and the effectiveness of the treatment program in reducing mortality. A study of HIV-infected children from Cambodia evaluated mortality prior to and after ART initiation and found that the majority of deaths occurred before starting ART [6]. Similar to this study, the majority of deaths occurred within the first three months after enrollment and among children who were eligible for ART. The overall mortality rate for children who never started ART was 7.7 per 100 person-years and was higher than the mortality rate among children receiving ART (2.0 per 100 person-years). Another study among 192 Ugandan children who were ineligible for ART found that 19% progressed to a WHO stage 3 or 4 event, death or ART eligibility after a median of 605 days [13]. Most events, three of which were deaths, occurred within the first year of follow-up. Predictors of progression included lower CD4^+^ T-cell percentage, higher viral load, and younger age. Anemia was marginally associated with progression but WAZ was not associated. Additional studies reported that 1% of eligible and 3% of ineligible children in Zambia died prior to ART initiation [3], and that 12.3% of ART-naïve children compared to 9.5% of children on ART in Mozambique died during follow-up [12]. Similar trends of high pre-ART mortality in the first few months of follow-up and similar risk factors were also found in studies of HIV-infected adults [5], [7], [8], [9], [10]. In this study, the cumulative mortality prior to ART initiation was 13.3% and was comparable to the cumulative mortality of 14.4% among children initiating ART in this cohort [15]. In addition, the mortality rate in the first three months after study enrollment among children eligible for ART was comparable to the mortality rate in the first three months after ART initiation (unpublished data). While mortality rates differ between programs depending on the characteristics of the population and the rigor of ascertainment of deaths, these estimates indicate that a significant proportion of children entering care are dying before they initiate ART.

The majority of deaths occurred in the first few months after study enrollment and during the period of preparation for ART. The median time to ART initiation among eligible children was 2.1 months. Other studies reported an average time of 4.7 months to ART initiation among children in Cambodia [6], 0.9 months among children in Lusaka, Zambia [3], one month among adults in South Africa [7], and 4.3 months among adults in The Gambia [11]. This period of preparation takes several clinic or home visits to ensure caregivers are prepared to administer life-long medication and be responsible for maintaining high adherence in their children. This process can take longer in rural areas as transportation and travel distance pose challenges, and necessitate longer intervals between visits and synchronization of visits with other family members [3]. Additional delays in ART initiation can lengthen this period, and can be due to treatment for concurrent tuberculosis or other illnesses, insufficient human resources (primarily in the early years of a treatment program), incorrect clinical or immunological staging by physicians, and social problems in the family, such as lack of transportation, food insecurity, no legal guardian, history of suboptimal adherence, lack of disclosure of child's status to another adult, denial of child's status or need for ART and ill health of the caregiver [6], [21]. Similar reasons for delay were identified for 26.7% of children eligible for ART at study enrollment in this study. Many of these delays were warranted to stabilize the child's health or ensure that the family was prepared for the burden of administering ART to the child. However, some delays also were attributable to clinician judgment and could potentially be prevented through continued training.

In this study, 77.8% of children who died were eligible for ART and were therefore characterized by a younger age and later stage of disease progression, including higher WHO stage, lower CD4^+^ T-cell percentage, lower WAZ, and lower hemoglobin. These characteristics were well-established risk factors for mortality among HIV-infected children in sub-Saharan Africa prior to the availability of ART [22], [23], [24]. Many children enroll in treatment programs when eligible for ART [3], as did 60% of children in this study. Identification of risk factors for mortality that could distinguish those children at risk of early death might allow for interventions to halt disease progression and prevent death. In this study, risk factors for mortality among children eligible for ART included younger age, severe undernutrition and severe anemia. Interestingly, CD4^+^ T-cell percentage was not predictive of early mortality, although the median CD4^+^ T-cell percentage was relatively high in this group of children. These risk factors are similar to risk factors for early mortality among children receiving ART [3], [25], [26], [27]. As a result of the late stage of disease at study enrollment, most deaths occurred within the first few months of follow-up before children could prepare for and start ART. Consequently, continued efforts are needed to promote testing of infants and children so that HIV-infected children can be identified and enrolled into care at an earlier stage of disease. If children could initiate treatment at lower levels of immunosuppression and with fewer concurrent illnesses, many of these pre-ART deaths could be prevented.

There were several limitations to this study. First, the follow-up time was relatively short and the number of observed deaths was low, particularly among children ineligible for ART, which limited our ability to evaluate risk factors for mortality in this group. Second, study enrollment was used as a proxy for clinic enrollment, as children had only been seen in the clinic for a short duration prior to study enrollment. The reported mortality rates may, therefore, underestimate the true mortality rate in this population, as deaths may have occurred soon after clinic enrollment which would not have been captured. However, the loss to follow-up was low and it is unlikely that many deaths were not reported. The mortality rate is likely to be accurate for the group of children surviving long enough to enroll in the study. Lastly, eligibility at study enrollment was defined retrospectively based on immunologic and clinical criteria. Some misclassification of eligibility status is likely to have occurred, as children with undernutrition in this setting who are initially classified as WHO stage 3 or 4 are not considered eligible for ART if they gain weight on nutritional support and treatment. In addition, some children were missing information on WHO stage or CD4^+^ T-cell percentage at study enrollment and were classified based on immunologic or clinical criteria alone.

In summary, a significant number of HIV-infected children enrolled in treatment programs die before initiating ART as a result of late-stage disease. These results further underscore the need to increase efforts to identify HIV-infected children at an earlier age and stage of disease so they can enroll in HIV care and treatment programs prior to becoming eligible for ART. In this way, they and their family can be prepared to initiate life-long therapy and receive the full benefits of treatment.
